# Evaluation of the In Vitro Blood–Brain Barrier Transport of *Ferula persica* L. Bioactive Compounds

**DOI:** 10.3390/ijms26168017

**Published:** 2025-08-19

**Authors:** Pouya Mohammadnezhad, Alberto Valdés, Melis Cokdinleyen, Jose A. Mendiola, Alejandro Cifuentes

**Affiliations:** Foodomics Laboratory, CIAL, CSIC-UAM, Nicolas Cabrera 9, 28049 Madrid, Spain; p.mohammadnezhad@cial.uam-csic.es (P.M.); melis.cokdinleyen@cial.uam-csic.es (M.C.); j.mendiola@csic.es (J.A.M.); a.cifuentes@csic.es (A.C.)

**Keywords:** bioactive compounds, blood–brain barrier, flavonoid sulfate, LC/GC-q-TOF-MS/MS, neuroprotection

## Abstract

Species of the *Ferula* genus are known for their traditional medicinal applications against diverse illnesses. Our previous study was the first to suggest the cholinesterase inhibitory activity of *Ferula persica* L. However, the neuroprotective efficacy of therapeutic molecules is often limited by their ability to cross the blood–brain barrier (BBB) and reach the brain. In the present study, the BBB permeability of the main molecules present in the aerial parts and roots of *F. persica* L. extracted under optimum conditions was assessed using two well-established methods: the parallel artificial membrane permeability assay (PAMPA) and the HBMEC cell culture in vitro model. The results demonstrated a high permeability of several neuroprotective compounds, such as apigenin, diosmetin, and α-cyperone. Additionally, the neuroprotective potential of *F. persica* extracts was evaluated using SH-SY5Y neuron-like cells exposed to different insults, including oxidative stress (H_2_O_2_), excitotoxicity (L-glutamate), and Aβ1-42 peptide toxicity. However, none of the obtained extracts provided significant protection. This study highlights the importance of in vitro cell culture models for a better understanding of BBB permeability mechanisms and reports the tentative identification of newly formed sulfated metabolites derived from the metabolism of ferulic acid, apigenin, and diosmetin by HBMEC cells.

## 1. Introduction

Neurodegenerative diseases include both hereditary and sporadic disorders that progressively and irreversibly lead to the loss of neural cells, ultimately impairing the proper functioning of the nervous system [1]. Alzheimer’s disease (AD) is a neurodegenerative disorder primarily associated with aging, and it is the leading cause of dementia, accounting for 60–80% of cases [2,3,4]. In 2015, AD affected approximately 47.4 million people worldwide, with projections estimating 75.6 million cases by 2030 and 135.4 million by 2050, highlighting its significant social and economic impact [5]. Histopathological features of AD include aggregates of hyperphosphorylated tau protein and amyloid-beta (Aβ) peptide, which form intracellular neurofibrillary tangles (NFTs) and extracellular senile plaques (SPs), respectively. Additional key hallmarks include a decline in the cholinergic system, an increase in oxidative stress, and significant neuroinflammation [6,7]. Available palliative treatments, such as galantamine and rivastigmine drugs, primarily aim to increase acetylcholine levels in the synaptic cleft by inhibiting acetylcholinesterase (AChE) and butyrylcholinesterase (BChE) enzymes. However, no definitive cure for AD is currently available [5].

One of the major challenges in developing effective treatments against AD is the limited potential of therapeutic agents to reach the brain. The blood–brain barrier (BBB), a dynamic interface between the central nervous system (CNS) and the systemic circulation, represents a significant obstacle to the delivery of bioactive compounds, effectively preventing neurotherapeutic agents from reaching their intended targets. The BBB comprises tightly linked endothelial cells that form junctional complexes, greatly limiting paracellular molecular transport. As a result, nearly 98% of small neurotherapeutic molecules and almost all large molecules cannot cross the BBB [8]. Therefore, demonstrating the permeability of synthetic drugs or natural bioactive molecules across the BBB is essential to ensure the delivery of these compounds to the brain tissues and to validate their potential neuroprotective effects [9]. In this context, different in vitro and in vivo models have been utilized to study the transport of molecules from natural products across the BBB [10]. Among the in vitro models, the parallel artificial membrane permeability assay for the BBB evaluation (PAMPA-BBB) is a high-throughput, non-cell-based permeation test introduced by [11]. This method is a low-cost, reproducible, and high-throughput permeability test, making it ideal for evaluating novel drugs or therapeutic bioactive compounds. The PAMPA-BBB assay is useful to assess the transcellular passive diffusion across the BBB, making it particularly applicable for preliminary compound screening. However, drug molecules cross the BBB via pathways different than passive diffusion, including receptor-mediated transcytosis, cell-mediated transcytosis, transporter-mediated transcytosis, and adsorptive mediated transcytosis [12]. These pathways can be better mimicked using in vitro cell culture models, such as human brain capillary endothelial cell lines, consisting of confluent monolayers of cells cultured on semi-permeable inserts. This configuration establishes two distinct chambers: an upper (luminal) chamber simulating the blood side, and a lower (basolateral) chamber representing the brain side [13,14]. Among the different models, the human brain microvascular endothelial cells (HBMECs), cultured alone or in combination with other cell lines, is a suitable cell line in terms of barrier tightness and paracellular permeability [15,16].

Apart from the different cell models to evaluate the BBB permeability, the human neuroblastoma SH-SY5Y is another cell line widely employed as an in vitro model of AD [17,18]. This cell line expresses a number of human-specific proteins and protein isoforms, and the differentiation of the cells into neurons entails a number of specific events, including a decrease in proliferation rate, formation and extension of neurites, and expression of mature neuronal markers [19]. SH-SY5Y cells have been extensively applied to study the neuroprotective potential of several natural compounds and extracts, particularly regarding oxidative stress [20] and neurodegenerative mechanisms [21,22].

Given the growing interest in identifying neuroprotective agents from natural sources, the integration of such in vitro models offers an effective approach for evaluating the efficacy of bioactive compounds from medicinal plants. For decades, many researchers have focused on traditional plant-based medicine, contributing to the discovery of natural products with antioxidant and anti-AChE activities. Among various natural sources, the *Ferula* genus (Apiaceae) is a perennial herb indigenous to Iran that has been used for the treatment of various organ illnesses [23], and some reports have highlighted its use for the treatment of neurological disorders such as AD [24]. However, the beneficial properties of this plant have been scarcely investigated concerning AD hallmarks. In this regard, we have previously evaluated and demonstrated the antioxidant, anti-cholinergic (AChE and BChE enzyme inhibition), and anti-inflammatory (LOX enzyme inhibition) capacities of different extracts obtained from the aerial parts and roots of *F. persica* L. using green extraction methods [25]. The results demonstrated that the extract obtained from the aerial parts using ethyl acetate (EtAc) (79%) and cyclopentyl methyl ether (CPME) (21%) at 180 °C and the extract obtained from the roots using 100% CPME at 180 °C were the most promising due to their strong bioactivities, and they were chemically characterized using analytical techniques (HPLC-Q-TOF-MS/MS and GC-Q-TOF-MS). As a result, it was determined that these extracts contain several flavonoids and sesquiterpenoids already reported as good antioxidant, anti-inflammatory, and neuroprotective molecules, some of which could potentially cross the BBB and reach the brain [26,27,28]. However, the BBB permeability of the main compounds of these complex extracts, as well as their neuroprotective capacity in cell culture models, remains to be evaluated.

Another important aspect to consider when evaluating the beneficial properties of natural compounds (using in vivo and/or in vitro cell culture models) is that metabolic processes can modify the native compounds, and therefore, new molecules can emerge. For instance, some studies have shown that natural compounds such as polyphenols can undergo different metabolic reactions (glucuronidation/sulfation conjugation or methylation) [10,29] that can change their potential biological effects, such as neuroprotection [13] or BBB permeability [30]. For instance, methylation combined with sulfation enhanced the transport of gallic acid and catechol derivatives, but not of pyrogallol derivatives [13]; sulfated gallocatechin gallate and sulfated catechin gallate were identified in the basolateral medium after BBB transport [30]. Complementary to these observations, there is a growing biochemical and chemical interest in sulfated flavonoids due to their potential biological effects, such as anticoagulant, antiviral, antimicrobial, antioxidant, or anti-inflammatory agents [31,32]. However, and although the transport of flavonoids across the BBB is widely studied [29], the presence and/or permeability of modified flavonoids, such as sulfated diosmetin and sulfated apigenin, has never been reported.

Based on the above information, the main objectives of the present work were the assessment of the BBB permeability of the main molecules present in the extracts obtained from the aerial parts and roots of *F. persica* L. by using two well-established methods (the PAMPA-BBB assay and the HBMEC in vitro cell culture model) and the evaluation of the neuroprotective effects of these extracts against different insults related to AD by using a neuron-like cell culture model (SH-SY5Y cells). Finally, this study reports, for the first time, the presence of different sulfated metabolites derived from the metabolism of ferulic acid, apigenin, and diosmetin by HBMEC cells, highlighting their different BBB permeabilities.

## 2. Results and Discussion

### 2.1. Evaluation of the Blood–Brain Barrier Permeability of F. persica Compounds Using the PAMPA-BBB Model

The first major objective of the present work was the assessment of the BBB permeability of the main molecules present in the extracts obtained from the aerial parts and roots of *F. persica* L. (AerExt and RootExt). This evaluation represents a critical approach for identifying neuroprotective compounds capable of accessing the CNS.

In our previous research article, we provided the most comprehensive and exhaustive chemical characterization of *F. persica* samples to date, which resulted from combining the use of pressurized liquid extraction (PLE) technology with different solvents, advanced HPLC-Q-TOF-MS/MS and GC-Q-TOF-MS instrumentation, and updated metabolite databases. A total of 222 compounds belonging to different subclasses (hydroxycoumarins, flavones, O-methylated flavonoids, methoxyphenols, monoterpenoids, and sesquiterpenoids) were successfully identified [25]. The present study further investigated the permeability of these compounds across the PAMPA-BBB using the same instrumentation (HPLC-Q-TOF-MS/MS and GC-Q-TOF-MS). In this context, several molecular factors, including the molecular weight (MW), lipophilicity (measured as the oil/water partition coefficient, log *p*), and topological polar surface area (TPSA) significantly influence the diffusion of compounds across the BBB. Additionally, other molecular properties, such as the number of hydrogen bond donors, the octanol/water partition coefficient at a defined pH, the Hansen polarity, and the ionization constant (pKa), can also be considered when evaluating permeability [33,34,35].

As shown in Table 1, 69 compounds belonging to relevant chemical subclasses were quantified in the donor plate of the PAMPA-BBB model, derived from both AerExt and RootExt. Most of these compounds (49) were quantified by HPLC-MS/MS, while 20 were quantified by GC-MS. Complementary to the quantitative analysis, the identities of 15 compounds (quinic acid, 2,3-dihydroxybenzoic acid, 4-hydroxybenzaldehyde, apigenin, luteolin, luteolin 7-glucoside, apigenin 7-glucoside, hesperidin, ethyl caffeate, ferulic acid, caffeic acid, 4-coumaric acid, 6,7-dihydroxycoumarin, diosmetin, and nobiletin) were confirmed by injecting standards (they are marked with an asterisk in Table 1 and Table 2, and their molecular structure can be found in Supplementary Figure S1). It has to be noted that a compound previously identified as benzoic acid was now confirmed to be 4-hydroxybenzaldehyde. Among the 69 quantified compounds in the donor plate, the PAMPA-BBB log Pe values could be calculated for 46 compounds, as the other compounds could not be quantified in the acceptor plate. In addition, other compounds could not be detected in the donor plate due to their low initial concentration in the extracts (e.g., ethyl caffeate in RootExt), which explains the different BBB permeability results obtained for the same compound in AerExt and RootExt extracts. The subclasses that account for more compounds crossing the PAMPA-BBB were carbonyl compounds, flavonoids (including flavones, methylated and some glycosylated forms), hydroxycinnamic acids and their derivatives, hydroxycoumarins, methoxyphenols, and sesquiterpenoids, although not all the compounds belonging to these subclasses could be quantified in the acceptor plate. Some anisoles (β-asarone and elemicin) and benzoic acids and their derivatives (2,3-dihydroxybenzoic acid and 3-formylsalicylic acid) also crossed the BBB, but none of the lignan glycosides could. In total, 38 compounds crossed the PAMPA-BBB from AerExt, while 32 compounds crossed from RootExt. As can be seen in Table 1, all compounds with a MW > 470 Da could not cross the BBB, which agrees with previously published articles [35]. However, different behavior was observed for compounds with a MW below 470 Da: many hydroxycinnamic acids and methylated flavonoids could cross the BBB, while many sesquiterpenoids could not. In this context, several authors have proposed different guidelines to explain the accessibility of natural compounds to the BBB. For example, Agatonovic-Kustrin et al. (2020) suggested that high log *p* values enhance BBB penetration due to increased lipophilicity [36]. In contrast, Hitchcock (2008) and Waterhouse (2003) reported that optimal BBB permeability is observed in molecules with log *p* values between 0 and 3 [35,37]. These last values agree well with our findings, as most of the compounds crossing the BBB have log *p* values between 1.5 and 3.6, while many sesquiterpenoids (not present in the acceptor plate) have log *p* values above 3.6. Also, we observed that the transport of flavonoids across the BBB decreased as the number of glycosides and hydroxyl groups in their structures increased. In particular, the aglycone forms, luteolin and apigenin, and other methylated flavonoids (diosmetin and nobiletin) exhibited high log Pe values, indicating better permeability compared with their glycosylated counterparts (luteolin 7-glucoside and apigenin 7-glucoside) and other glycosylated flavonoids (isoquercetrin and hesperidin). These results were consistent with the findings of Sánchez-Martínez et al. (2022) and Yang et al. (2014) [38,39]. Moreover, and from a neuroprotective perspective, the presence of glycosylated moieties in the molecular structure of flavonoids might not only reduce their bioactive potential [40] but also limit their BBB permeability due to their high MW and TPSA and low log *p* value. Finally, a TPSA of 90 Å^2^ has been suggested as an upper threshold for the BBB permeability [35]. Our findings partially agreed with that value, but some compounds with a TPSA higher than 90 Å^2^ were able to efficiently cross the PAMPA-BBB (such as luteolin and diosmetin). The combination of these factors (including high MW, elevated TPSA, and low log *p* values), together with the low concentration of some compounds in the original extract (such as chrysoeriol or kaempferol), likely contributed to the absence and non-quantification of several compounds in the acceptor plate of AerExt and RootExt (Table 1). Conversely, catechol, luteolin, apigenin, diosmetin, pentalenic acid, norharman, 7-hydroxy-6-methoxycoumarin, isofraxidin, 8-hydroxyageraphorone, nobiletin, and (–)-caryophyllene oxide demonstrated good BBB permeability, as evidenced by their quantification in the acceptor plate after both extracts. Among them, diosmetin, apigenin, nobiletin, and luteolin exhibited the highest potential for BBB permeation in AerExt, with PAMPA-BBB log Pe values of −3.83, −4.01, −4.31, and −4.58, respectively. These results agree well with previous studies demonstrating the high BBB permeability of flavonoids [41] and their neuroprotective potential [42,43,44,45].

In our previous study, to identify the compounds potentially responsible for the observed in vitro neuroprotective activity, a Pearson’s correlation analysis was performed between compound abundance and ROS scavenging and AChE inhibitory activities. That study revealed some compounds significantly correlated with both ROS scavenging capacity and AChE inhibition, among which nobiletin and isofraxidin were identified. As presented above, these compounds have also demonstrated high PAMPA-BBB permeability in AerExt and RootExt, and both compounds have already shown interesting neuroprotective properties [46,47,48,49]. Other low molecular weight compounds, such as ferulic acid, have been also reported to exhibit favorable BBB permeability, as demonstrated in both in vivo studies and PAMPA-BBB models [50]. It is worth noting that two β-carbolines tentatively identified in our previous research, harman and norharman, have been reported to exhibit antioxidant, anti-inflammatory, and neuroprotective effects. Both compounds were detected in AerExt and showed high BBB permeability. Furthermore, Ref. [51] reported that 3,4-dihydroxyacetophenone, a compound capable of crossing the BBB, positively correlated with thinned peach extracts’ neuroprotective potential. This finding aligns with our results, as this compound was quantified in AerExt and demonstrated significant BBB permeability, with a log Pe value of –4.76. Another compound with multiple health beneficial and potential neuroprotective effects is chrysoeriol [52], which was identified in the donor plate of AerExt (but was not quantified in the acceptor plate due to its low concentration in the extract).

All these findings, combined with the previous results from the in vitro enzymatic assays, suggest that many phytochemicals identified in *F. persica* L. extracts have the potential to cross the BBB and exert different neuroprotective effects, and therefore, we further investigated some of these properties using cell culture in vitro models.

### 2.2. In Vitro Toxicity and Cell Barrier Integrity Assay of F. persica Extracts in HBMEC Cells

The second major objective of the present work was to investigate and validate the BBB permeability of bioactive compounds from AerExt and RootExt using a complex in vitro cell culture model. However, prior to this evaluation, it was essential to evaluate if there was any damage to the BBB monolayer caused by the extracts. Therefore, a viability assay was first performed on HBMEC cells to determine the safe concentrations of AerExt and RootExt to be used. The tested concentrations were 10, 20, and 40 µg mL^−1^.

**Figure 1:**
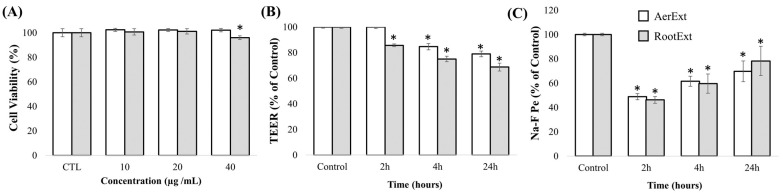
(**A**) Cell viability (%) of HBMEC cells after exposure to different concentrations of *F. persica* aerial part (AerExt) and root (RootExt) extracts for 24 h. (**B**) HBMEC transendothelial electrical resistance (TEER) changes after AerExt (40 µg mL^−1^) and RootExt (20 µg mL^−1^) treatments at different incubation times (2, 4, and 24 h). (**C**) HBMEC sodium fluorescein (Na-F) paracellular permeability after AerExt (40 µg mL^−1^) and RootExt (20 µg mL^−1^) treatments at different incubation times (2, 4, and 24 h). Asterisks (*) denote statistical differences after T-test between control and extract-treated cells at different incubation times, *p*  <  0.05.

As can be observed in Figure 1A, AerExt did not affect the cell viability at the highest concentration tested (40 μg mL^−1^), while RootExt slightly decreased the viability to 90% (at 40 μg mL^−1^) compared with the control group, but not at 20 μg mL^−1^.

Based on these results, the highest non-toxic concentrations (40 µg mL^−1^ for AerExt and 20 µg mL^−1^ for RootExt) were selected to evaluate the BBB integrity and the transport assays. To evaluate the BBB integrity after the different incubation times (2, 4, and 24 h), two methods were applied: TEER measurement and Na-F paracellular permeability. The results showed that when AerExt was applied, TEER values were close to the control value at 2 h, but a significant decrease was observed after 4 and 24 h (Figure 1B). Similarly, RootExt caused a significant decrease in TEER values at all time points (2, 4, and 24 h). However, despite the decrease in TEER values, the Na-F permeation analyses revealed that both extracts resulted in lower Na-F permeation compared with the control at all time points (2, 4, and 24 h), suggesting that the cell monolayer integrity was not compromised (Figure 1C).

### 2.3. Evaluation of F. persica Compound Transport Across the BBB Endothelium

Once the absence of toxicity of AerExt or RootExt was confirmed on HBMEC cells, the transport of the compounds across the BBB monolayer at different incubation times (2, 4, and 24 h) was evaluated using the same instrumentation (HPLC-Q-TOF-MS/MS and GC-Q-TOF-MS) as previously used for the PAMPA-BBB assay. The results are presented in Table 2, where 28 compounds were quantified in the apical compartment once the results of AerExt and RootExt were combined. As can be observed, this number was lower compared with the PAMPA-BBB results (69 compounds), and no compounds could be quantified by using GC-MS instrumentation. This reduction can be explained by the fact that the working concentration used within the cells was 40 µg mL^−1^ (for AerExt) and 20 µg mL^−1^ (for RootExt), which is 125 and 250 times lower than the concentration used in the PAMPA-BBB assay (5 mg mL^−1^), therefore hampering the detection of the least-abundant compounds. Despite this aspect, 15 compounds exhibited measurable transport efficiency (Te) combining both extracts: 12 compounds were quantified at 2, 4, and 24 h after AerExt, while 4, 4, and 7 compounds were quantified after RootExt at 2, 4, and 24 h, respectively. The higher number of compounds permeating from AerExt might be a consequence of the different compound compositions between both extracts (as shown in [25]) but also due to the higher concentration used for this matrix (40 µg mL^−1^) compared with the 20 µg mL^−1^ used for RootExt. Moreover, although the number of compounds that could cross the BBB was similar at the different incubation times after AerExt, most of the compounds detected in the basolateral chamber exhibited their highest Te after 24 h of incubation. These aspects may also reflect different interactions between AerExt and RootExt extracts with the endothelial cells (such as membrane binding or cellular uptake before translocation), and not only passive diffusion mechanisms.

HBMEC results were then compared with those obtained from the PAMPA-BBB assay, and three main categories of compounds could be differentiated. The first category included compounds with calculated log Pe in the PAMPA-BBB that could also cross the HBMEC-BBB. Among these compounds, apigenin and diosmetin were the compounds with the highest log Pe values that exerted the highest permeability through the HBMEC-BBB model after AerExt. For both compounds, the Te dramatically increased after 24 h, being up to 300% in the case of apigenin. This high value might be explained by the high permeability of apigenin (log Pe = −4.01) combined with the intracellular metabolism of different apigenin derivatives (such as apigenin 7-glucoside) or other related flavonoids (such as luteolin, which possesses only a one-hydroxyl difference with respect to apigenin) that completely disappeared from the apical compartment, potentially contributing to the increase in apigenin in the basolateral compartment. Another interesting compound that Te increased to 53% in AerExt and to 187% in RootExt was α-cyperone. Its good permeability, together with the results observed in our previous study [25] and in other studies [53], supports the status of α-cyperone as a molecule with promising neuroprotective potential. Other compounds with good BBB potential (−4.3 > log Pe > −5.5) from the PAMPA-BBB assay that could also cross the HBMEC-BBB after AerExt were β-asarone, loliolide, ferulic acid, 7-hydroxy-6-methoxycoumarin, (–)-caryophyllene oxide, and norharman, whose Te values were modest and slightly varied during the incubation time (in the case of ferulic acid, it increased from 2% after 2 h to 20% after 24 h). However, in the case of RootExt, the Te values of 2-hydroxy-5-methoxybenzaldehyde, 4-hydroxybenzaldehyde, 2,3-dihydroxybenzoic acid, isofraxidin, ophiopogonoside A, and 8-hydroxyageraphorone highly increased after 24 h.

The second category included compounds with calculated log Pe in the PAMPA-BBB assay that could not cross the HBMEC-BBB, such as protocatechuic aldehyde, 1,2,4-benzenetriol, apigenin 7-glucoside, 2,5-dihydroxycinnamic acid, 4-methoxysalicylaldehyde, luteolin, and nobiletin. Among them, it is interesting to note that nobiletin and luteolin were compounds with good PAMPA-BBB potential (log Pe of −4.31 and −4.58, respectively) and already reported as good neuroprotective molecules [44]. In addition, luteolin was one of the most abundant compounds in AerExt [25].

The third category included compounds with low BBB potential in the PAMPA-BBB that could not cross the HBMEC-BBB model. This category included glycosylated compounds (such as hesperidin, olivil glucoside isomers, and syringaresinol diglucoside), but also 4-coumaric acid, ophiopogonoside A, and chrysoeriol. All these compounds were quantified in the apical but not in the basolateral compartment after AerExt.

**Figure 2:**
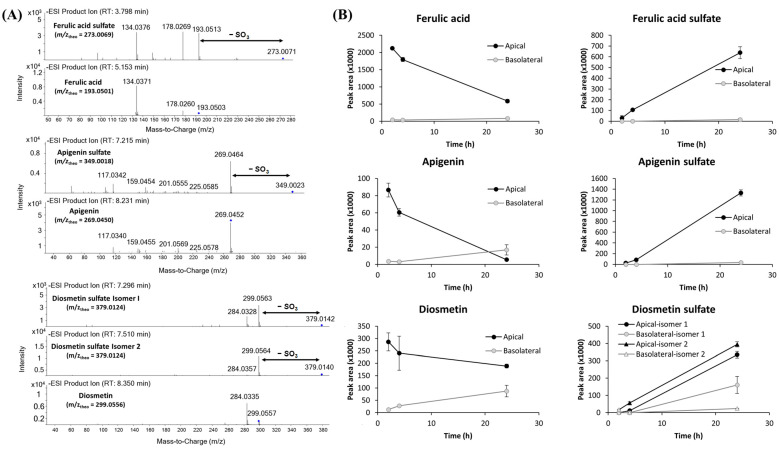
(**A**) HPLC–MS/MS data and (**B**) relative quantification (peak areas) of newly formed metabolites (ferulic acid sulfate, apigenin sulfate, and two diosmetin sulfate isomers) and their non-sulfated counterparts (ferulic acid, apigenin, and diosmetin) appearing in the apical (black) and lower (gray) compartments of HBMEC cell experiment after AerExt (40 µg mL^−1^) treatment at different incubation times (2, 4, and 24 h).

As previously commented, the transport percentage of bioactive molecules across the endothelium may be influenced by their metabolism within the cells. To identify possible cellular metabolites, the HPLC-MS/MS data for the upper and lower compartments (after AerExt and RootExt treatments) was searched against predicted theoretical masses and fragmentation patterns of methyl, sulfate, glutathione, and glucuronic acid conjugated metabolites. Based on these comparisons, four sulfated molecules (ferulic acid sulfate, apigenin sulfate, and two isomeric forms of diosmetin sulfate) were tentatively identified after AerExt treatment based on their exact mass and MS/MS fragmentation pattern (Figure 2A). As can be observed, the experimental *m/z* values perfectly matched the theoretical *m/z* values of the sulfated molecules, which, together with a neutral loss of 79.955 (–SO_3_), provide an *m/z* value corresponding to the non-sulfated form, a similar MS/MS fragmentation pattern compared with the non-sulfated form, and an earlier retention time (the sulfated molecules are more polar than the non-sulfated forms), allowed us to propose these identities. In this respect, it is interesting to note that none of these sulfated derivatives were identified in the original *F. persica* extracts [25], suggesting that sulfation occurred during the interaction of the extract with HBMEC cells. Based on these identifications, the relative abundance of these newly formed metabolites and their non-sulfated counterparts was assessed, which indicated that all of them followed similar time-dependent patterns (Figure 2B). Overall, the abundance in the apical compartment of the non-sulfated forms was decreased with time, while their abundance in the basolateral compartment was increased, indicating that extended exposure time allows for slow translocation processes across the endothelial barrier. Thus, prolonged incubation may facilitate passive diffusion for low-permeability compounds as well as active transport or intracellular metabolism followed by export. Additionally, interactions with cellular components, such as membrane binding, could contribute to the delayed presence of compounds on the brain side. However, the Te of these molecules was different at 24 h: 14.2% for ferulic acid, 46.3% for diosmetin, and 309.7% for apigenin, suggesting different transport mechanisms for each molecule. On the other hand, the abundance of the sulfated forms of ferulic acid, apigenin, and diosmetin continuously increased in both the apical and the basolateral compartments. In all cases, the abundance in the apical compartment was higher than in the basolateral compartment, being almost 40 times higher for ferulic acid sulfate (Te = 2.3%) and apigenin sulfate (Te = 2.7%). However, it was interesting to observe that the relative (apical/basolateral) abundance of both diosmetin sulfate isomers was higher than the other two molecules, with Te = 47.8% (diosmetin sulfate-Isomer 1), and Te = 5.9% (diosmetin sulfate-Isomer 2). Similar results have been observed in previous studies, where different sulfated polyphenols (such as gallocatechin gallate-O-sulfate, catechin gallate-O-sulfate, 4-O-methylgallic acid-3-O-sulfate, pyrogallol-O-sulfate, resveratrol sulfate, or feruloyl-4-O-sulfate) have been identified in different in vitro or in vivo BBB models [10]. Since sulfonation increases the polarity and aqueous solubility of compounds, several types of membrane transport have been considered for their transport, such as ATP-binding cassette transporters (ABCs), organic anion transporters (OATs) and organic anion-transporting polypeptides (OATPs) [50]. However, this is the first time the BBB permeabilities of apigenin sulfate and diosmetin sulfate are reported, highlighting the role of brain endothelial cells not only as a physical barrier but also as active participants in the metabolism and transport of compounds. Moreover, we hypothesize that the newly formed molecules specifically correspond to ferulic acid-4-O-sulfate, apigenin acid-7-O-sulfate, diosmetin-7-O-sulfate, and diosmetin-3’-O-sulfate (as they are the most abundant isomers in nature), but further experiments are needed to validate these results.

**Table 2:** Transport efficiency (Te) values of bioactive compounds identified in *Ferula persica* L. aerial parts (AerExt) and roots (RootExt) after HBMEC cell culture in vitro assay.

Analytical Platform	Compound	RT (min)	Molecular Formula	Monoisotopic Mass	Subclass	MW	log *p*	TPSA	AerExt			RootExt		
									Te (%) ± SD			Te (%) ± SD		
									2 h	4 h	24 h	2 h	4 h	24 h
LC-MS(+)	β-asarone	9.032	C_12_H_16_O_3_	208.10994	Anisoles	208.25	3.0	27.7	7.5 ± 2.0	15.0 ± 7.3	15.5 ± 1.0	–	–	–
LC-MS(−)	1,2,4-benzenetriol	1.464	C_6_H_6_O_3_	126.03169	Benzenetriols and derivatives	126.11	1.5	60.7	–	–	–	n.q.	n.q.	n.q.
LC-MS(+)	Loliolide	5.481	C_11_H_16_O_3_	196.10994	Benzofurans	196.24	1.0	46.5	6.2 ± 0.7	12.1 ± 0.8	12.4 ± 0.8	–	–	–
LC-MS(−) *	2,3-dihydroxybenzoic acid	3.572	C_7_H_6_O_4_	154.02661	BAs and derivatives	154.12	1.5	57.5	17.5 ± 3.5	9.6 ± 0.6	14.0 ± 3.9	21.4 ± 5.3	23.9 ± 4.4	214.1 ± 45.4
LC-MS(−) *	4-hydroxybenzaldehyde	3.771	C_7_H_6_O_2_	122.03677	Carbonyl compounds	122.12	1.4	37.3	23.3 ± 3.1	18.5 ± 0.3	49.4 ± 5.9	62.1 ± 21.9	57.5 ± 1.7	184.6 ± 23.0
LC-MS(−)	Protocatechuic aldehyde	3.013	C_7_H_6_O_3_	138.03169	Carbonyl compounds	138.12	1.3	57.5	n.q.	n.q.	n.q.	n.q.	n.q.	–
LC-MS(−) *	Apigenin	8.215	C_15_H_10_O_5_	270.05282	Flavones	270.05	1.7	90.9	4.2 ± 0.9	5.1 ± 0.3	309.7 ± 93.0	–	–	–
LC-MS(−) *	Luteolin	7.553	C_15_H_10_O_6_	286.04774	Flavones	286.24	1.4	111.1	n.q.	n.q.	n.q.	–	–	–
LC-MS(−) *	Hesperidin	6.254	C_28_H_34_O_15_	610.18977	Flavonoid glycosides	610.6	−1.1	234.3	n.q.	n.q.	n.q.	n.q.	n.q.	n.q.
LC-MS(−) *	Apigenin 7-glucoside	6.281	C_21_H_20_O_10_	432.10565	Flavonoid glycosides	432.38	−0.1	170.1	n.q.	n.q.	–	–	–	–
LC-MS(−) *	Ferulic acid	5.167	C_10_H_10_O_4_	194.05791	HCAs and derivatives	194.18	1.5	66.8	1.9 ± 0.2	1.9 ± 0.2	19.8 ± 10.3	n.q.	n.q.	n.q.
LC-MS(−)	2,5-dihydroxycinnamic acid	4.068	C_9_H_8_O_4_	180.04226	HCAs and derivatives	180.15	1.2	77.8	n.d.	n.d.	n.d.	n.q.	n.q.	n.q.
LC-MS(−) *	4-coumaric acid	4.671	C_9_H_8_O_3_	164.04734	HCAs and derivatives	164.04	1.5	57.5	n.q.	n.q.	n.q.	n.q.	n.q.	n.q.
LC-MS(+)	7-hydroxy-6-methoxycoumarin	5.114	C_10_H_8_O_4_	192.04226	Hydroxycoumarins	192.16	1.5	59.7	0.6 ± 0.2	1.9 ± 0.1	6.1 ± 1.5	n.q.	n.q.	n.q.
LC-MS(+)	Isofraxidin	5.410	C_11_H_10_O_5_	222.05282	Hydroxycoumarins	222.19	1.5	68.9	n.q.	n.q.	n.q.	n.q.	n.q.	30.5 ± 6.6
LC-MS(+)	Olivil glucoside isomer 1	4.298	C_26_H_34_O_12_	538.20503	Lignan glycosides	538.50	0.1	187.8	n.q.	n.q.	n.q.	–	–	–
LC-MS(+)	Olivil glucoside isomer 2	4.299	C_26_H_34_O_12_	538.20503	Lignan glycosides	538.50	0.1	187.8	n.q.	n.q.	n.q.	–	–	–
LC-MS(+)	Syringaresinol diglucoside	5.117	C_34_H_46_O_18_	742.26841	Lignan glycosides	742.74	−1.4	254.1	n.q.	n.q.	n.q.	–	–	–
LC-MS(−)	2-hydroxy-5-methoxybenzaldehyde	4.152	C_8_H_8_O_3_	152.04734	Methoxyphenols	152.15	1.8	46.5	18.8 ± 2.3	9.1 ± 1.2	11.0 ± 1.3	31.8 ± 8.0	36.3 ± 10.9	164.8 ± 21.0
LC-MS(−)	4-methoxysalicylaldehyde	4.843	C_8_H_8_O_3_	168.04226	Methoxyphenols	152.15	1.5	46.5	n.q.	n.q.	n.q.	n.q.	–	–
LC-MS(−) *	Diosmetin	8.355	C_16_H_12_O_6_	300.06339	O-methylated flavonoids	300.26	1.7	100.1	4.5 ± 0.2	12.1 ± 3.7	46.3 ± 14.1	–	–	–
LC-MS(−)	Chrysoeriol	8.683	C_16_H_12_O_6_	300.06339	O-methylated flavonoids	300.26	1.7	100.1	n.q.	n.q.	n.q.	–	–	–
LC-MS(+) *	Nobiletin	8.976	C_21_H_22_O_8_	402.13147	O-methylated flavonoids	402.4	3.0	85.6	n.q.	n.q.	n.q.	n.q.	n.q.	n.q.
LC-MS(+)	Norharman	4.120	C_11_H_8_N_2_	168.06875	Pyridoindoles	168.2	3.2	41.9	2.3 ± 0.1	5.3 ± 1.4	4.1 ± 0.2	–	–	–
LC-MS(+)	α-cyperone	9.871	C_15_H_22_O	218.16707	Sesquiterpenoids	218.33	3.8	17.1	12.2 ± 0.1	25.1 ± 1.4	56.5 ± 6.5	15.4 ± 2.6	20.1 ± 12.0	187.3 ± 12.5
LC-MS(+)	(-)-caryophyllene oxide	9.492	C_15_H_24_O	220.18272	Sesquiterpenoids	220.35	3.6	12.5	4.3 ± 0.1	8.6 ± 0.6	8.8 ± 0.7	n.q.	n.q.	n.q.
LC-MS(+)	8-hydroxyageraphorone	8.088	C_15_H_24_O_2_	236.17763	Sesquiterpenoids	236.35	2.9	37.3	n.q.	n.q.	n.q.	n.q.	n.q.	124.7 ± 16.9
LC-MS(+)	Ophiopogonoside A	6.235	C_21_H_38_O_8_	418.25667	Terpene glycosides	418.50	0.4	139.8	–	–	–	n.q.	n.q.	66.0 ± 3.2

RT: retention time; MW: molecular weight; log *p*: oil/water partition coefficient; TPSA: topological polar surface area; Te: transport efficiency; LC-MS(+): HPLC-MS/MS with Electrospray Ionization in positive mode (+); LC-MS (−): HPLC-MS/MS with Electrospray Ionization in negative mode (−); HCAs: hydroxycinnamic acids; BAs: benzoic acids; n.q.: not quantified in the basolateral compartment; – (not detected in the apical nor in the basolateral compartment). Asterisks (*) denote compounds confirmed using standards.

### 2.4. In Vitro Neuroprotective Potential of F. persica Extracts in SH-SY5Y Cells

**Figure 3:**
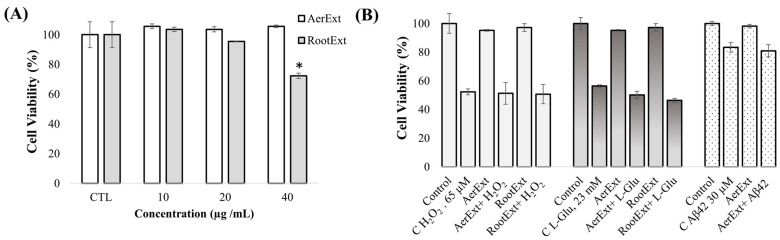
(**A**) Cell viability (%) of SH-SY5Y cells after exposure to different concentrations of *F. persica* aerial parts (AerExt) and roots (RootExt) extracts for 24 h (* denotes statistical differences between control and extract-treated cells, *p*  <  0.05). (**B**) Neuroprotective effects of AerExt (40 µg mL^−1^) and RootExt (20 µg mL^−1^) against the neurotoxic agents H_2_O_2_ (65 µM), L-glutamate (23 mM), and Aβ1-42 (30 µM) in differentiated SH-SY5Y cells. Non-treated cells were used as control, together with only extracts-treated cells at 40 µg mL^−1^ (AerExt) and 20 µg mL^−1^ (RootExt). The results are expressed as the mean (*n*  = 3)  ±  SD.

The final objective of the present study was to evaluate the neuroprotective effects of AerExt and RootExt against different insults related to AD. For that aim, neuroprotection against H_2_O_2_, L-glutamate, and Aβ1-42 was evaluated using a neuron-like cell culture model (SH-SY5Y cells). Firstly, in vitro toxicity assessment of AerExt and RootExt was evaluated at different concentrations. As can be seen in Figure 3A, none of the three concentrations tested for AerExt (10, 20, and 40 µg mL^−1^) significantly affected the viability of SH-SY5Y cells after 24 h, whereas RootExt was toxic at 40 µg mL^−1^, significantly decreasing cell viability to 72% (*p* < 0.05). Therefore, the highest non-toxic concentrations (40 µg mL^−1^ for AerExt and 20 µg mL^−1^ for RootExt) were selected to evaluate their neuroprotective effects.

To evaluate the neuroprotection capacity of the extracts against different insults, differentiated SH-SY5Y cells were seeded and incubated with non-toxic concentrations of extracts (40 µg mL^−1^ for AerExt and 20 µg mL^−1^ for RootExt) for 24 h. After that, the neurotoxic agents H_2_O_2_ or L-glutamate were added for another 24 h at 65 µM and 23 mM, respectively. Controls containing only cell growth medium were also included to determine the maximum cell viability. As can be observed in Figure 3B, H_2_O_2_ decreased the cell viability to 48% compared with the control group, but the pre-treatment of SH-SY5Y cells with AerExt or RootExt did not show any significant protection. A similar effect was observed for the protection against L-glutamate. At 23 mM, L-glutamate decreased the cell viability to 47% compared with control cells (Figure 3B), but no significant protection was observed when the cells were pre-treated with *F. persica* extracts. Since the neuroprotection capacity of AerExt was most promising due to its lack of toxicity at higher concentration (40 µg mL^−1^), its higher AChE and LOX inhibition capacity, and its higher content in potential bioactive compounds [25], its neuroprotection capacity against Aβ1-42 peptide (30 µM) was also tested. At this concentration, Aβ1-42 peptide decreased the cell viability to 83% compared with control cells, but no significant protection was observed when the cells were pre-treated with AerExt. All these results demonstrate that the concentration of potential bioactive compounds in *F. persica* extracts are not enough to protect against oxidative stress, excitotoxicity, or Aβ1-42 peptide toxicity. Despite these observations, and based on the neuroprotective potential reported for other sulfated polyphenols [13], the ability of HBMEC cells to conjugate the main flavonoids present in AerExt (apigenin and diosmetin) with sulfate, and the potential of these sulfated molecules to cross the BBB, new experiments are being designed to test their neuroprotective potential. However, sulfated flavonoids are not commercially available, and they are unstable and difficult to isolate from natural sources; therefore, they have to be specifically synthesized, purified, and chemically characterized before they are ready to be used for further in vitro experiments.

## 3. Materials and Methods

### 3.1. Chemicals and Reagents

SH-SY5Y and HBMEC cells were obtained from ATCC® (Rockville, MD, USA). Roswell Park Memorial Institute (RPMI) 1640 medium, fetal bovine serum (FBS), non-essential amino acids (NEAAs), minimal essential medium (MEM) vitamins, sodium pyruvate, L-glutamine, trypsin-EDTA, antibiotic solution (including penicillin and streptomycin), thiazolyl blue tetrazolium bromide (MTT), PBS, n-dodecane, porcine polar brain lipid (PBL), PAMPA-BBB 96-well donor plate (Cat. MAIPNTR10), PAMPA-BBB 96-well acceptor plate (Cat. MATRNPS50), sodium fluorescein (molecular weight, 376 Da), p-coumaric acid, caffeic acid, ferulic acid, quinic acid, hesperidin, and 4-hydroxybenzaldehyde were purchased from Sigma-Aldrich (St. Louis, MO, USA). Apigenin 7-O-glucoside, luteolin, apigenin, ethyl caffeate, 2,3-dihydroxybenzoic acid, and 6,7-dihydroxycoumarin were acquired from BLD Pharmatech (Reinbek, Germany). Luteolin 7-O-glucoside was purchased from Extrasynthese (Genay, France), diosmetin was acquired from Carbosynth Ltd. (Berkshire, UK), nobiletin was obtained from TCI Chemicals (Tokyo, Japan), and NuSerum IV was obtained from Corning Costar Corp (New York, NY, USA). Transwell cell culture chambers (12-well plates) with 3.0 μm pore size and rat tail collagen type I solution (CLS354236) were purchased from Corning Life Sciences (Tewksbury, MA, USA). Brain-derived neurotrophic factor (BDNF) and amyloid-beta 1–42 (Aβ1-42) peptide were obtained from Hello Bio (Dunshaughlin, Republic of Ireland), while retinoic acid was acquired from Glentham Life Sciences (Corsham-Wiltshire, UK). Light microscopy for monitoring cell cultures was performed using a Nikon Inverted Microscope (ECLIPSE, TE2000-U, Tokyo, Japan). The 96-well plates were all purchased from Costar, Corning, USA. Hank’s balanced salt solution (HBSS) was obtained from Lonza (Basel, Switzerland). LC-MS-grade acetonitrile (ACN), LC-MS-grade methanol and ethanol (EtOH), cyclopentyl methyl ether (CPME), and ethyl acetate (EtAc) were obtained from VWR Chemicals (Barcelona, Spain), whereas Milli-Q water was obtained from a Millipore system (Billerica, MA, USA). Formic acid was purchased from Fisher Scientific (Waltham, MA, USA).

### 3.2. Sample Preparation and Extraction Conditions

*Ferula persica* L., including its leaves, roots, twigs, and stems, was collected from the mountains near Shahmirzad, Iran. The plant parts were air-dried at room temperature and subsequently stored under refrigeration. For the experiments, the aerial parts (leaves, twigs, and flowers) and roots were homogenized and sieved to a particle size of 250–500 µm. *F. persica* aerial parts and roots extracts were obtained using an Accelerated Solvent Extractor (ASE 200, Dionex Corporation, Sunnyvale, CA, USA), according to our previous work [25]. Briefly, 1 g of *F. persica* aerial parts or roots was placed into an 11 mL extraction cell, and the extraction was carried out during 20 min at 10 MPa and 180 °C by using a mixture of EtAc (79%) and CPME (21%) for aerial parts (named AerExt) or 100% CPME for roots (named RootExt). These extracts were comprehensively characterized in our previous article by HPLC-Q-TOF-MS/MS and GC-Q-TOF-MS [25].

### 3.3. Parallel Artificial Membrane Permeability Assay—Blood–Brain Barrier

The permeability of bioactive compounds from AerExt and RootExt across the BBB was assessed using the PAMPA-BBB assay, following the methodology described by [11]. Firstly, the BBB solution was prepared by dissolving 8 mg of PBL and 4 mg of cholesterol in 600 μL of n-dodecane. The initial donor solution was prepared by mixing 1 mL of AerExt or RootExt (at 10 mg mL^−1^ in EtOH) with 1 mL of buffer (PBS, pH = 7.4, 10 mM). The filter membrane of the donor plate was coated with 5 μL of BBB solution. The acceptor plate was filled with 350 μL of buffer, and the donor plate was carefully placed on top of the acceptor plate to form a “sandwich-like” assembly. Subsequently, 350 μL of the donor solution was added to the donor plate, which was then covered and incubated at 37 °C for 4 h in the dark. After the incubation time, 300 μL of the solution was collected from each plate, transferred into separate vials, and evaporated using a SpeedVac system at 40 °C under 13 mbar pressure. The dried acceptor and donor solutions were dissolved in 50 μL of pure EtOH before being subjected to HPLC-Q-TOF-MS/MS and GC-Q-TOF-MS analysis. Permeability (P_e_) across the PAMPA-BBB of the bioactive compound was calculated according to Equation (1) [54], with modifications in concentration rates.

P_e_ = −ln[1 − C_A_(t)/C_equilibrium_]/(A × (1/V_D_ + 1/V_A_) × t) (1)

P_e_ refers to the permeability of the bioactive compound across the PAMPA-BBB in cm s^−1^, A is the effective filter area = 0.3 cm^2^ (given by the manufacturer), V_D_ is the donor well volume = 0.35 mL, V_A_ is the acceptor well volume = 0.35 mL, t is the incubation time (s) = 14,400, C_A_(t) is the peak area of the compound in the acceptor well at time t, and C_D_(t) is the peak area of the compound in the donor well at time t. C_equilibrium_ was calculated according to Equation (2):

C_equilibrium_ = [C_D_(t) × V_D_ + C_A_(t) × V_A_]/(V_D_ + V_A_) (2)

### 3.4. Cell Culture Assays in HBMEC Cells

#### 3.4.1. Toxicity Evaluation of *F. persica* Extracts 

HBMEC cells were cultured as described by [55]. Briefly, cells were grown in RPMI 1640-based medium with the addition of 10% FBS, 10% NuSerum IV, 1% NEAA, 1% MEM, 1 mM sodium pyruvate, 2 mM L-glutamine, and 1% antibiotic–antimycotic mixture. Cells were incubated at 37 °C in a humidified incubator with 5% CO_2_. Cells between passages 5 and 10 were used in the experiments. For toxicity analyses, HBMEC cells were seeded in 96-well plates pre-coated with rat tail type I collagen (100 μg mL^−1^) at a density of 2.5 × 10^4^ cells mL^−1^ in a volume of 100 μL each. After 24 h of cell attachment, AerExt or RootExt was added to the medium at different concentrations. The cells were incubated for another 24 h after this treatment. At the end of the incubation period, cell viability was determined using the MTT assay. To do so, the extract solutions were removed and 0.5 mg mL^−1^ MTT solution was added to each well. The cells were then incubated at 37 °C for 3 h, the MTT was carefully removed, and formazan crystals were dissolved in 100 µL of DMSO. The absorbance of the solution was measured at 570 nm wavelength using a plate reader (Cytation 5, BioTek Instruments, Winooski, VT, USA). Cell viability was calculated by comparing the absorbance values of treated cells against the control group (ethanol-treated cells). Ethanol concentration did not exceed 0.4% (*v*/*v*). All the experiments were performed in triplicate, and a T-test was applied between the treated and control conditions considering statistically significant differences when *p* < 0.05.

#### 3.4.2. Blood–Brain Barrier Transport Study of *F. persica* Extracts

After the toxicity evaluation, the HBMEC cell line was also used as a simplified in vitro model of the BBB endothelium. Cells were seeded at a density of 1.6 × 10^5^ cells/cm^2^ in 12-well Transwell^®^ plates (Corning Costar Corp., Corning, USA) with a pore diameter of 0.4 μm, pre-coated with type I rat tail collagen (100 μg mL^−1^) [55]. The culture medium was renewed every 2 days until a monolayer cell structure was formed (see Supplementary Figure S2), and transport experiments were initiated after 10 days. In transport experiments, the medium in the apical (upper) compartment of the Transwell system was removed, and 0.5 mL of AerExt or RootExt at a concentration of 40 μg mL^−1^ and 20 μg mL^−1^, respectively, was added to these compartments (applied in duplicate). Also, a medium containing 0.4% (*v*/*v*) ethanol was used as control group. The basolateral (lower) compartments were filled with 1.5 mL of fresh culture medium. The cells were incubated under these conditions for 2, 4, and 24 h.

At the end of the incubation periods, media samples were collected from both chambers and lyophilized using a freeze-drying system from Martin Christ (Osterode am Harz, Germany). The samples were stored at −80 °C until analysis. The collected samples were subjected to chemical analyses to evaluate the ability of bioactive compounds contained in the extracts to pass through the cell monolayer.

The transport efficiency (Te) of bioactive substances was calculated based on Equation (3), described by [56]:

Te (%) = C_Lower_/C_Upper_ × 100 (3)

This calculation was performed to understand whether the compound crosses the cell layer and how efficiently it is transported. Hence, C_Lower_ represents the peak area of the compound in the lower compartment, and C_Upper_ represents the peak area of the compound in the upper compartment.

#### 3.4.3. Cell Barrier Integrity

Transendothelial electrical resistance (TEER) measurement and paracellular permeability test using sodium fluorescein (Na-F) are two complementary analysis methods widely used to evaluate the structural and functional integrity of the cell monolayer in the BBB model [55]. Since these parameters reflect the tightness of intercellular junctions and endothelial permeability, they are critical to ensure the accuracy and reliability of the model. In the study, TEER values and Na-F diffusion measurements were performed to reveal the possible barrier-disrupting effects of AerExt or RootExt on the BBB model cells.

*Transendothelial electrical resistance (TEER)*

Transendothelial electrical resistance (TEER) analyses were performed using the EndOhm™ electrode system (World Precision Instruments, Sarasota, FL, USA) connected to the EVOMX resistance meter as previously described [55]. TEER data were recorded at four time points: 0 h (immediately before extract application) and at 2, 4, and 24 h after the application. The electrical resistance values of the blank inserts without cells and extracts were subtracted from each measurement as the background signal; then, the net resistance values were multiplied by the insert area (1.12 cm^2^), and the results were expressed in the standard unit ohm-square centimeter (Ω × cm^2^). These calculated electrical resistance values were compared with the mean values of the control group without extracts, the percentage change rates were determined, and a T-test was applied considering statistically significant differences when *p* < 0.05.

After completion of TEER measurements, the culture media in the upper (apical) and lower (basolateral) compartments were carefully collected and prepared for analysis to be used in subsequent transport experiments.

*Sodium fluorescein (Na-F) paracellular permeability*

The Na-F (sodium fluorescein) permeability test was performed as previously described [55]. This test was used to measure the paracellular permeability of the junctions between cells after the influence of the extracts. Once the TEER measurements and the collection of the cell culture medium were performed, the inserts containing the cells were washed twice with HBSS solution. These inserts were then placed into new 12-well plates containing 1.5 mL of HBSS solution, and 0.5 mL of 10 μg mL^−1^ Na-F dissolved in the same buffer was added to the upper compartments to be in contact with the cells. The inserts were transferred to new wells containing HBSS solution after incubation at 37 °C for 20, 40, and 60 min, protected from light. At the end of the incubation periods, the inserts were removed and the Na-F concentration in the lower chamber were analyzed using a fluorescence spectrophotometer (Cytation 5, BioTek Instruments, Winooski, VT, USA; excitation: 460 nm and emission: 515 nm). The Na-F flux through the cell layers without extracts (control) and through the cell-free inserts (blanks) was also measured. Finally, the transendothelial permeability coefficient (PS_e_) was calculated as previously described by [57]. This coefficient quantifies how much Na-F is transported across the cell layer and the permeability of the cell layer. Briefly, the clearance volume of Na-F permeation into the lower compartment was determined using Equation (4):

Clearance (μL) = C_Lower_ × V_Lower_ × C_Upper_^−1^ (4)

where C_Lower_ and C_upper_ represent the Na-F concentration in the lower and upper compartments, respectively, and V_Lower_ represents the volume of the lower compartment (1.5 mL). Then, the average volume cleared was plotted versus time to obtain the clearance slopes, and the permeability of the endothelial monolayer (PS_e_) was calculated using a linear regression considering the clearance slopes after the extracts treatments (PS_time−plotted_) and the blank inserts without cells (PS_insert_), following Equation (5):

PS_e_^−1^ = PS_time-plotted_^−1^ − PS_insert_^−1^ (5)

PS_e_ divided by the surface area (1.12 cm^2^) generates the endothelial permeability coefficient [Pe (cm/s)]. This value was compared with the control group without the addition of the extracts, expressing the change in permeability of the cell layer as a percentage and applying a T-test to identify statistically significant differences (*p* < 0.05).

### 3.5. Cell Culture Assays in SH-SY5Y Cells

#### 3.5.1. Toxicity Evaluation of *F. persica* Extracts

SH-SY5Y neuroblastoma cells were cultured and differentiated in the same conditions as previously described [21]. Cells were grown in DMEM/F12 medium containing 10% FBS, 100 U mL^−1^ penicillin, 100 µg mL^−1^ streptomycin, and 250 ng mL^−1^ antimycotic in 5% CO_2_ and 95% humidity at 37 °C incubation conditions. The culture medium was replaced with a fresh medium every three days. Differentiation was performed for 7 days to obtain a neuron-like phenotype, and the phenotype development was confirmed by morphological evaluations under the microscope. After differentiation, cells were trypsinized and reseeded in 24-well plates at 42,000 cells/cm^2^ density. After incubating the cells for 24 h, AerExt or RootExt was added to the medium at different concentrations, and the cells were incubated with the extracts for another 24 h. Cell viability was assessed using the MTT protocol as described above. All experiments were performed with three independent replicates.

#### 3.5.2. Neuroprotection Evaluation of *F. persica* Extracts

To investigate the neuroprotective potential of AerExt or RootExt, oxidative damage (H_2_O_2_), excitotoxic stress (L-glutamate), and Aβ1-42 toxicity were induced in cells and evaluated as described by [58]. Briefly, SH-SY5Y cells were plated as described above, and 24 h after cell attachment, cells were pre-treated with non-toxic concentrations of AerExt or RootExt (40 μg mL^−1^ for AerExt; 20 μg mL^−1^ for RootExt) for 24 h. In the control group, DMEM/F12 cell medium supplemented with 1% FBS, 100 U mL^−1^ penicillin, 100 μg mL^−1^ streptomycin, 250 ng mL^−1^ antimycotic, and 0.4% ethanol was used. The next day, cells were incubated with either cell medium alone (control), H_2_O_2_ (65 μM), L-glutamate (23 mM), or Aβ1-42 (30 μM) for 24 h. Cell viability was assessed by MTT assay at the end of the experiments. All the experiments were repeated three times, and a T-test was applied between the treated and control conditions considering statistically significant differences when *p* < 0.05.

### 3.6. Quantification of F. persica Compounds in Barrier Transport Assays

#### 3.6.1. Compound Extraction

The samples obtained from the upper and lower compartments of the transport assays performed in HBMEC cells were extracted to recover and concentrate the bioactive compounds. For this purpose, 0.2 mL of cold water (H_2_O) was added to the samples, and the samples were vortexed for 10 s. Then, 0.8 mL of cold methanol was added and vortexed again for 10 s. Then, the mixture was centrifuged at 14,000 rpm for 5 min, and the supernatant was carefully transferred to a 1.5 mL Eppendorf tube and dried in a SpeedVac device at 40 °C and 13 bar pressure. Finally, the dried samples were resuspended in 25 μL of pure ethanol for their subsequent analysis.

#### 3.6.2. High Performance Liquid Chromatography–Tandem Mass Spectrometry (HPLC-Q-TOF-MS/MS) and Gas Chromatography–Mass Spectrometry (GC-Q-TOF-MS) Analysis

The analysis of the samples obtained from the PAMPA-BBB assay and the HBMEC cells experiments was carried out using the same conditions and instrumentation as in our previous study [25]. For HPLC-Q-TOF MS/MS analysis, aliquots of 2 μL were injected into an HPLC model 1290 coupled to a Q-TOF series 6540 (Agilent Technologies, Waldbronn, Germany). For GC-Q-TOF MS analysis, aliquots of 1 μL were injected with a split ratio of 1:10 into a GC model 7890B coupled to a Q-TOF series 7200 (Agilent Technologies, Waldbronn, Germany). The HPLC-MS and GC-MS chromatograms were processed using the Agilent Mass Hunter Qualitative software (version B.10.0) to verify the retention time and exact mass of the compounds previously reported by us [25] and to extract their peak area to calculate the BBB permeability. Additional information for each bioactive compound, such as log *p* and TPSA, was obtained from the PubChem (https://pubchem.ncbi.nlm.nih.gov/ (accessed on 20 May 2025)) and SwissADME (http://www.swissadme.ch/ (accessed on 20 May 2025)) websites, respectively. ClassyFire Batch by Fiehn Lab website was used for the structural classification of compounds into their respective subclasses (https://cfb.fiehnlab.ucdavis.edu/ (accessed on 20 May 2025)).

## 4. Conclusions

In conclusion, we investigated the ability of potential bioactive compounds from extracts of *F. persica* L. aerial parts and roots to cross the BBB using two complementary in vitro approaches: the PAMPA-BBB assay and the HBMEC cell culture model. The results showed that different carbonyl compounds, flavonoids, hydroxycinnamic acids, hydroxycoumarins, methoxyphenols, and sesquiterpenoids could cross the artificial PAMPA-BBB model. However, some of these compounds (such as nobiletin or luteolin) failed to permeate HBMEC cells, highlighting the importance of complementary in vitro cell culture assays for a better understanding of the BBB permeability mechanisms. On the other hand, other already demonstrated neuroprotective compounds (such as apigenin, diosmetin, or α-cyperone) were able to cross the BBB in the in vitro cell culture model (mainly after 24 h of incubation), suggesting that active and transcellular mechanisms play an important role in combination with passive diffusion. Despite these results, the concentration of potential bioactive compounds in *F. persica* extracts were not enough to protect SH-SY5Y neuroblastoma cells against oxidative stress, excitotoxicity, or Aβ1-42 peptide toxicity. Complementary to the compounds originally present in *F. persica* extracts, four newly formed sulfated derivatives (ferulic acid sulfate, apigenin sulfate, and two diosmetin sulfate isomers) were tentatively identified in the apical compartment of HBMEC cells and could also cross the BBB with different efficiency, and future experiments are being designed to synthesize, purify, and evaluate their neuroprotective potential.

## Figures and Tables

**Table 1:** Blood–brain barrier (BBB) permeability values of bioactive compounds identified in *Ferula persica* L. aerial parts (AerExt) and roots (RootExt) extracts after parallel artificial membrane permeability assay (PAMPA).

Analytical Platform	Compound	RT (min)	Molecular Formula	Monoisotopic Mass	Subclass	MW	log *p*	TPSA	AerExt			RootExt		
									log Pe (cm^−1^)	RSD (%)	Cross BBB Potential	log Pe (cm^−1^)	RSD (%)	Cross BBB Potential
LC-MS(−) *	Quinic acid	0.694	C_7_H_12_O_6_	192.06339	Alcohols and polyols	192.17	−2.4	118.2	−6.51	1.27	+	n.d.		
LC-MS(+)	β-asarone	8.912	C_12_H_16_O_3_	208.10994	Anisoles	208.25	3.0	27.7	−4.86	1.40	++	n.q.		–
GC-MS	Elemicin	11.731	C_12_H_16_O_3_	208.10990	Anisoles	208.25	2.5	74.6	−5.28	1.54	++	−4.68	0.65	++
LC-MS(−) *	2,3-dihydroxybenzoic acid	3.342	C_7_H_6_O_4_	154.02661	BAs and derivatives	154.12	1.2	77.8	n.q.		–	−5.42	2.53	++
LC-MS(−)	3-formylsalicylic acid	2.848	C_8_H_6_O_4_	166.02661	BAs and derivatives	166.13	1.0	74.6	n.d.			−5.50	1.70	++
LC-MS(−)	1,2,4-benzenetriol	1.365	C_6_H_6_O_3_	126.03169	Benzenetriols and derivatives	126.11	1.5	60.7	n.q.		–	−5.19	1.60	++
LC-MS(−)	Catechol	2.380	C_6_H_6_O_2_	110.03678	Benzenediols	110.11	0.9	40.5	−4.88	0.90	++	−4.70	1.60	++
GC-MS	Myristicin	11.444	C_11_H_12_O_3_	192.07860	Benzodioxoles	192.21	2.9	40.5	n.d.			n.q.		–
LC-MS(+)	Loliolide	5.162	C_11_H_16_O_3_	196.10994	Benzofurans	196.24	1.0	46.5	−4.98	2.17	++	n.d.		
LC-MS(−) *	4-hydroxybenzaldehyde	3.445	C_7_H_6_O_2_	122.03677	Carbonyl compounds	122.12	1.4	37.3	−4.52	0.46	++	−4.33	1.76	+++
LC-MS(−)	Protocatechuic aldehyde	2.745	C_7_H_6_O_3_	138.03169	Carbonyl compounds	138.12	1.3	57.5	−5.05	0.94	++	−4.85	1.40	++
LC-MS(−)	3,4-dihydroxyacetophenone	3.324	C_8_H_8_O_3_	152.04734	Carbonyl compounds	152.15	1.5	57.5	n.d.			−4.76	1.59	++
LC-MS(+)	9,19-cyclolanost-25-ene-3,24-diol	9.900	C_30_H_50_O_2_	442.38108	Cycloartanols and derivatives	442.70	8.6	40.5	−5.60	1.25	+	−5.79	1.93	+
LC-MS(−) *	Apigenin	8.074	C_15_H_10_O_5_	270.05282	Flavones	270.24	1.7	90.9	−4.01	1.07	+++	n.q.		–
LC-MS(−) *	Luteolin	7.268	C_15_H_10_O_6_	286.04774	Flavones	286.24	1.4	111.1	−4.58	0.83	++	−4.68	0.72	++
LC-MS(−)	Hispidulin 4′-glucoside	6.302	C_22_H_22_O_11_	462.11621	Flavonoid glycosides	462.40	0.8	179.3	−6.14	2.04	+	n.d.		
LC-MS(−) *	Luteolin 7-glucoside	5.489	C_21_H_20_O_11_	448.10056	Flavonoid glycosides	448.40	0.5	190.3	−6.92	1.30	+	n.q.		–
LC-MS(−) *	Apigenin 7-glucoside	6.063	C_21_H_20_O_10_	432.10565	Flavonoid glycosides	432.40	−0.1	170.1	−7.34	3.38	+	n.q.		–
LC-MS(−)	Isoquercetrin	5.446	C_21_H_20_O_12_	464.09548	Flavonoid glycosides	464.40	−0.2	210.5	n.q.		–	n.q.		–
LC-MS(−) *	Hesperidin	6.050	C_28_H_34_O_15_	610.18977	Flavonoid glycosides	610.60	−1.1	234.3	n.q.		–	n.q.		–
LC-MS(−)	Kaempferol 3-O-(3″,4″-di-O-acetyl-a-L-rhamnopyranoside)	7.878	C_25_H_24_O_12_	516.12678	Flavonoid glycosides	516.40	−0.9	186.1	n.q.		–	n.d.		
LC-MS(+)	Harman	4.273	C_12_H_10_N_2_	182.08440	Harmala alkaloids	182.22	3.6	28.7	−4.62	0.51	++	n.d.		
LC-MS(−) *	Ethyl caffeate	7.188	C_11_H_12_O_4_	208.07356	HCAs and derivatives	208.21	2.6	66.8	−4.20	3.11	+++	n.d.		
LC-MS(−) *	Ferulic acid	4.905	C_10_H_10_O_4_	194.05791	HCAs and derivatives	194.18	1.5	66.8	−5.13	0.97	++	n.q.		–
LC-MS(−) *	Caffeic acid	3.591	C_9_H_8_O_4_	180.04226	HCAs and derivatives	180.16	1.2	77.8	−5.16	1.89	++	n.q.		–
LC-MS(−)	2,5-dihydroxycinnamic acid	3.820	C_9_H_8_O_4_	180.04226	HCAs and derivatives	180.16	1.2	77.8	−5.68	3.34	++	−5.22	1.61	++
LC-MS(−)	4-caffeoylquinic acid lactone	4.477	C_16_H_16_O_8_	336.08500	HCAs and derivatives	164.16	0.4	133.5	−5.81	0.56	+	−5.42	2.08	++
LC-MS(−) *	4-coumaric acid	4.400	C_9_H_8_O_3_	164.04734	HCAs and derivatives	336.29	1.5	57.5	n.q.		–	n.q.		–
LC-MS(+)	7-hydroxy-6-methoxycoumarin	4.885	C_10_H_8_O_4_	192.04226	Hydroxycoumarins	192.17	1.5	66.8	−4.66	1.13	++	−4.53	1.33	++
LC-MS(+)	Fraxidin	5.468	C_11_H_10_O_5_	222.05282	Hydroxycoumarins	222.19	1.5	68.9	−4.82	0.41	++	−4.81	1.03	++
LC-MS(+)	Isofraxidin	5.142	C_11_H_10_O_5_	222.05282	Hydroxycoumarins	222.19	1.5	68.9	−5.20	0.38	++	−5.03	2.17	++
LC-MS(−) *	6,7-dihydroxycoumarin	3.528	C_9_H_6_O_4_	178.02661	Hydroxycoumarins	178.14	1.2	70.7	−5.60	0.72	+	n.q.		–
LC-MS(+)	6″-O-acetylgenistin	7.644	C_23_H_22_O_11_	474.11621	Isoflavonoid O-glycosides	474.40	0.9	176.1	n.q.		–	n.d.		
LC-MS(+)	Olivil glucoside isomer 1	4.230	C_26_H_34_O_12_	538.20503	Lignan glycosides	538.50	0.1	187.8	n.q.		–	n.d.		
LC-MS(+)	Olivil glucoside isomer 2	4.230	C_26_H_34_O_12_	538.20503	Lignan glycosides	538.50	0.1	187.8	n.q.		–	n.d.		
LC-MS(+)	Syringaresinol glucoside	5.997	C_28_H_36_O_13_	580.21559	Lignan glycosides	580.60	−1.4	254.1	n.q.		–	n.q.		–
LC-MS(+)	Syringaresinol diglucoside	5.008	C_34_H_46_O_18_	742.26841	Lignan glycosides	742.70	−1.4	254.1	n.q.		–	n.q.		–
LC-MS(−)	2-hydroxy-5-methoxybenzaldehyde	3.857	C_8_H_8_O_3_	152.04734	Methoxyphenols	152.15	1.8	46.5	−4.71	0.65	++	−4.40	1.27	+++
GC-MS	4-vinylguaiacol	8.757	C_9_H_10_O_2_	150.0681	Methoxyphenols	150.17	2.4	46.5	−6.04	2.03	+	−5.36	4.47	++
LC-MS(-)	4-methoxysalicylaldehyde	4.586	C_8_H_8_O_3_	168.04226	Methoxyphenols	152.15	1.5	46.5	n.q.		–	−3.95	3.50	+++
GC-MS	trans-sinapyl alcohol	16.498	C_11_H_14_O_4_	210.08920	Methoxyphenols	210.23	1.3	66.8	n.d.			−6.04	3.10	+
LC-MS(−) *	Diosmetin	8.214	C_16_H_12_O_6_	300.06339	O-methylated flavonoids	300.26	1.7	100.1	−3.83	3.27	+++	−4.17	1.41	+++
LC-MS(+) *	Nobiletin	8.915	C_21_H_22_O_8_	402.13147	O-methylated flavonoids	402.40	3.0	85.6	−4.31	3.44	+++	−4.31	1.75	+++
LC-MS(−)	Chrysoeriol	8.582	C_16_H_12_O_6_	300.06339	O-methylated flavonoids	300.26	1.7	100.1	n.q.		–	n.d.		
LC-MS(+)	3,7-epoxycaryophyllan-6-One	7.431	C_15_H_24_O_2_	236.17763	Oxepanes	236.35	2.8	26.3	−4.98	0.52	++	n.d.		
LC-MS(+)	Norharman	3.794	C_11_H_8_N_2_	168.06875	Pyridoindoles	168.19	3.2	41.9	−4.05	0.83	+++	−4.67	8.83	++
LC-MS(+)	8-hydroxyageraphorone	7.814	C_15_H_24_O_2_	236.17763	Sesquiterpenoids	236.35	2.9	37.3	−4.65	0.91	++	−4.23	1.65	+++
LC-MS(+)	(–)-caryophyllene oxide	9.365	C_15_H_24_O	220.18272	Sesquiterpenoids	220.35	3.6	12.5	−4.69	0.64	++	−4.31	0.43	+++
LC-MS(−)	Pentalenic acid	9.946	C_15_H_22_O_3_	250.15689	Sesquiterpenoids	250.33	2.7	57.5	−4.84	1.18	++	−4.97	0.89	++
GC-MS	Farnesyl acetate	14.979	C_17_H_28_O_2_	264.20890	Sesquiterpenoids	264.40	5.3	210.5	−5.25	2.02	++	n.d.		
GC-MS	Valerianol	12.973	C_15_H_26_O	222.19840	Sesquiterpenoids	222.37	3.5	77.8	−5.35	1.45	++	−5.59	5.60	+
GC-MS	γ-eudesmol	12.709	C_15_H_26_O	222.19840	Sesquiterpenoids	222.37	3.7	77.8	−5.88	2.70	+	−5.69	6.85	+
GC-MS	epi-γ-eudesmol	13.049	C_15_H_26_O	222.19840	Sesquiterpenoids	222.37	2.9	46.5	−5.89	2.15	+	−5.81	3.89	+
GC-MS	α-bisabolol	13.405	C_15_H_26_O	222.19840	Sesquiterpenoids	222.37	1.3	133.5	−5.94	2.83	+	−5.75	6.23	+
LC-MS(+)	α-cyperone	9.734	C_15_H_22_O	218.16707	Sesquiterpenoids	218.33	3.8	17.1	−5.08	0.6	++	−5.16	0.29	++
GC-MS	γ-elemene	10.220	C_15_H_24_	204.18780	Sesquiterpenoids	204.35	5.4	29.5	n.q.		–	n.q.		–
GC-MS	(–)-aristolene	10.260	C_15_H_24_	204.18780	Sesquiterpenoids	204.35	4.7	118.2	n.q.		–	n.q.		–
GC-MS	α-humulene	10.550	C_15_H_24_	204.18780	Sesquiterpenoids	204.35	4.5	60.7	n.q.		–	n.q.		–
GC-MS	Selina-3,7(11)-diene	11.626	C_15_H_24_	204.18780	Sesquiterpenoids	204.35	4.4	57.5	n.q.		–	n.q.		–
GC-MS	(E)-nerolidol	11.730	C_15_H_26_O	222.19840	Sesquiterpenoids	222.37	4.6	57.5	n.q.		–	n.q.		–
GC-MS	Rosifoliol	12.308	C_15_H_26_O	222.19840	Sesquiterpenoids	222.37	3.9	77.8	n.q.		–	n.q.		–
GC-MS	Guaiol	12.382	C_15_H_26_O	222.19840	Sesquiterpenoids	222.37	3.1	37.3	n.q.		–	n.q.		–
GC-MS	α-epi-7-epi-5-eudesmol	12.507	C_15_H_26_O	222.19840	Sesquiterpenoids	222.37	3.4	70.7	n.q.		–	n.q.		–
GC-MS	Agarospirol	12.848	C_15_H_26_O	222.19840	Sesquiterpenoids	222.37	3.7	77.8	n.q.		–	n.q.		–
GC-MS	Bulnesol	13.189	C_15_H_26_O	222.19840	Sesquiterpenoids	222.37	3.8	57.5	n.q.		–	n.q.		–
GC-MS	Guaiol acetate	13.914	C_17_H_28_O_2_	264.20890	Sesquiterpenoids	264.40	3.6	46.5	n.q.		–	−5.05	1.36	++
LC-MS(+)	Ophiopogonoside A	6.047	C_21_H_38_O_8_	418.25667	Terpene glycosides	418.50	0.4	139.8	n.q.		–	n.q.		–
LC-MS(+)	4-methyl-5-thiazoleethanol	1.654	C_6_H_9_NOS	143.04048	Thiazoles	143.21	0.8	61.4	n.d.			−5.04	1.25	++
LC-MS(+)	Protopanaxadiol	10.603	C_30_H_52_O_3_	460.39165	Triterpenoids	460.70	7.2	60.7	n.q.		–	n.q.		–

RT: retention time; MW: molecular weight; log *p*: oil/water partition coefficient; TPSA: topological polar surface area; Pe: permeability; LC-MS(+): HPLC-MS/MS with Electrospray Ionization in positive mode (+); LC-MS(−): HPLC-MS/MS with Electrospray Ionization in negative mode (−); GC-MS: GC-MS with Electron Impact ionization mode; HCAs: hydroxycinnamic acids; BAs: benzoic acids; n.d.: not detected in the donor nor in the acceptor plate; n.q.: not quantified in the acceptor plate. Cross BBB potential: – (not quantified in acceptor); + (log Pe < −6.5); ++ (−6.5 < log Pe < −5.5), +++, (−5.5 < log Pe < −4.5). Asterisks (*) denote compounds confirmed using standards.

## Data Availability

Data contained within the article.

## Supplementary Materials

The following supporting information can be downloaded at: https://www.mdpi.com/article/10.3390/ijms26168017/s1.
