# Supplementary material for: Evaluation of the In Vitro Blood–Brain Barrier Transport of Ferula persica L. Bioactive Compounds
Source: Int J Mol Sci. 2025 Aug 19;26(16):8017. doi: 10.3390/ijms26168017 (PMC12386606; doi:10.3390/ijms26168017)
Supplement: Supplementary file 1 [file ijms-26-08017-s001.zip › ijms-3776159-supplementary.pdf]

Supplementary Figures

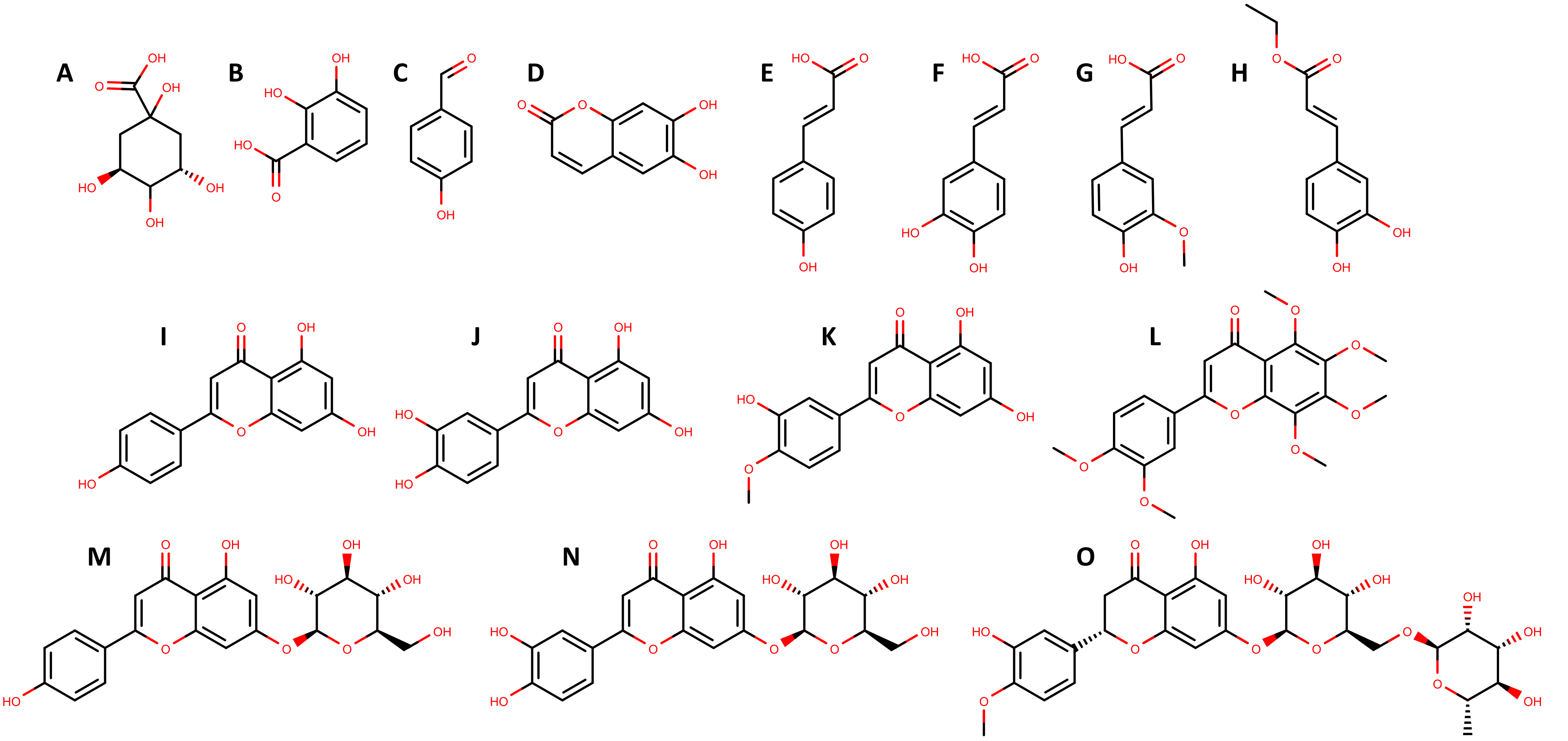

Figure S1. Molecular structure of compounds confirmed with standards: **A**, quinic acid; **B**, 2,3-dihydroxybenzoic acid; **C**, 4-hydroxybenzaldehyde; **D**, 6,7-dihydroxycoumarin; **E**, 4-coumaric acid; **F**, caffeic acid; **G**, ferulic acid; **H**, ethyl caffeate; **I**, apigenin; **J**, luteolin; **K**, diosmetin; **L**, nobiletin; **M**, apigenin 7-glucoside; **N**, luteolin 7-glucoside; **O**, hesperidin.

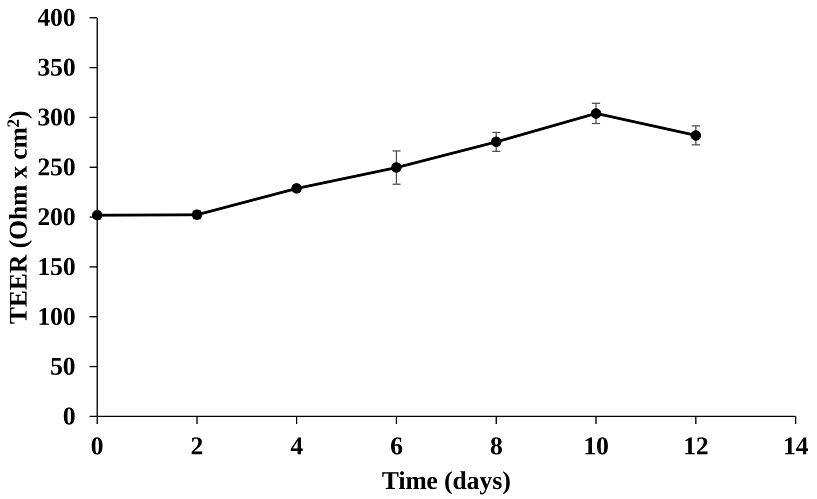

Figure S2. Transendothelial electrical resistance (TEER) was measured on HBMEC cells and plotted against time (days). Measurements were performed before changing the medium. One representative experiment is shown with mean ± SD from six wells. The blank insert without cells presented a mean value of 200 Ω × cm^2.
